# A case report of severe *Mycoplasma pneumoniae* pneumonia complicated by plastic bronchitis and Kawasaki disease

**DOI:** 10.1186/s12879-025-12063-2

**Published:** 2025-11-25

**Authors:** Hongjia Chen, Wanmin Xia, Yi Peng, Yijie Huang

**Affiliations:** Department of Pediatric Respiratory Medicine, School of Medicine, Chengdu Women’s and Children’s Central Hospital, University of Electronic Science and Technology of China, Chengdu, China

**Keywords:** Macrolide-resistant *Mycoplasma pneumoniae*, Plastic bronchitis, Kawasaki disease, Children

## Abstract

**Background:**

We report the first documented case of macrolide-resistant *Mycoplasma pneumoniae* (MRMP) infection simultaneously complicated by both plastic bronchitis (PB) and Kawasaki disease (KD), expanding our understanding of severe MRMP-associated immune dysregulation.

**Case presentation:**

A 7-year-old male presented with persistent high fever (39.8 °C), paroxysmal cough, and mucocutaneous manifestations. Diagnosis was established through multiple modalities: chest CT revealed bilateral consolidation with segmental airway narrowing; bronchoscopy demonstrated characteristic bronchial casts with focal mucosal necrosis; echocardiography showed right coronary artery dilation (Z-score + 2.334); and targeted next-generation sequencing (tNGS) identified MRMP with the A2063G mutation, alongside Streptococcus pneumoniae and Staphylococcus aureus co-infections. Treatment included oral doxycycline after macrolide failure, high-dose IVIG (2 g/kg), methylprednisolone (3 mg/kg/day), and therapeutic bronchoscopy. Complete resolution of coronary dilation and respiratory symptoms was achieved by the one-month follow-up.

**Conclusions:**

This unprecedented case demonstrates how MRMP infection can trigger simultaneous, severe immune-mediated complications through shared inflammatory pathways. In regions with high MRMP prevalence (> 90%), clinicians should maintain vigilance for atypical manifestations in refractory pneumonia. Early bronchoscopy and tNGS for comprehensive pathogen identification are essential, while combined therapy with appropriate alternative antibiotics, corticosteroids, and IVIG can effectively manage these complex cases.

**Clinical trial number:**

Not applicable.

The global spread of macrolide-resistant *Mycoplasma pneumoniae* (MRMP) has posed severe challenges to the clinical management of community-acquired pneumonia (CAP) in children, significantly compromising the therapeutic efficacy of conventional macrolide antibiotic treatment [1, 2]. This challenge is particularly acute in China, where recent large-scale epidemiological studies have confirmed that MRMP prevalence rates have consistently exceeded 90%, with some regions reaching 93.02% by May 2024, transforming this from a clinical concern into a major regional public health issue [1, 3, 4]. Growing evidence indicates that such high prevalence rates of MRMP infection are directly associated with more severe clinical courses and higher rates of pulmonary and extrapulmonary complications, imposing substantial burdens on healthcare systems [5, 6].

The core pathophysiological mechanisms driving these severe complications are believed to be post-infectious hyperinflammation and immune dysregulation [7, 8]. This uncontrolled immune response represents a key risk factor for triggering immune-mediated diseases such as plastic bronchitis (PB), Kawasaki disease (KD), or concurrent manifestations of both conditions [9, 10]. However, while existing research has recognized that MRMP can cause individual severe complications, the simultaneous triggering of both PB and KD—two distinct types of severe immune-mediated diseases—within the same patient represents an extremely rare clinical phenomenon. The underlying immunological crosstalk and pathophysiological mechanisms remain enigmatic.

Therefore, this article aims to provide an in-depth analysis of the unique clinical presentations, diagnostic challenges, and successful therapeutic strategies through a pediatric case of pathologically confirmed MRMP infection with concurrent PB and KD. This report not only seeks to fill this critical knowledge gap in the existing literature but also hopes to provide valuable diagnostic insights and management experience for clinicians confronting multi-system, severe immune complications induced by MRMP.

## Case presentation (Fig. 1)

A 7-year-old male was admitted to our tertiary care center on his 6th day of illness (defined as Hospital Day 1) with a chief complaint of “cough for 5 days and fever for 3 days.” The illness began 5 days prior to admission with a non-productive, dry cough that progressed from intermittent to severe and paroxysmal. On the 3rd day of illness, he developed a high fever of 39.5 °C accompanied by chills, which responded only transiently to antipyretics. Concurrently, he presented with conjunctival injection and dry, erythematous lips. At a local hospital, he received three days of intravenous cefuroxime and one day of intravenous azithromycin without significant clinical improvement.

Upon admission (Hospital Day 1), his temperature was 39.8 °C, heart rate was 130 beats/min, and respiratory rate was 32 breaths/min. Physical examination revealed a conscious child with mild, bilateral non-exudative conjunctival injection and dry, fissured, erythematous lips. The remainder of the ENT examination was unremarkable. Multiple tender cervical lymph nodes were palpable, with the largest being an asymmetric node of approximately 3 cm on the right side. Lung auscultation revealed coarse breath sounds bilaterally, without audible rales or wheezing.

Laboratory results from the outside hospital included a chest X-ray (CXR) showing radiographic features of bronchopneumonia, with increased, thickened, and blurred lung markings and small patchy opacities along the bronchial distribution in the middle and lower lung fields. A complete blood count (CBC) showed: white blood cell (WBC) count 7.30 × 10⁹/L, hemoglobin 132 g/L, neutrophils 50.2%, lymphocytes 30.9%, and platelets 225 × 10⁹/L. The C-reactive protein (CRP) was 9.6 mg/L. A *Mycoplasma pneumoniae* (MP) antibody test was negative (method unspecified). Intravenous piperacillin and supportive care were initiated upon admission.

**Fig. 1:**
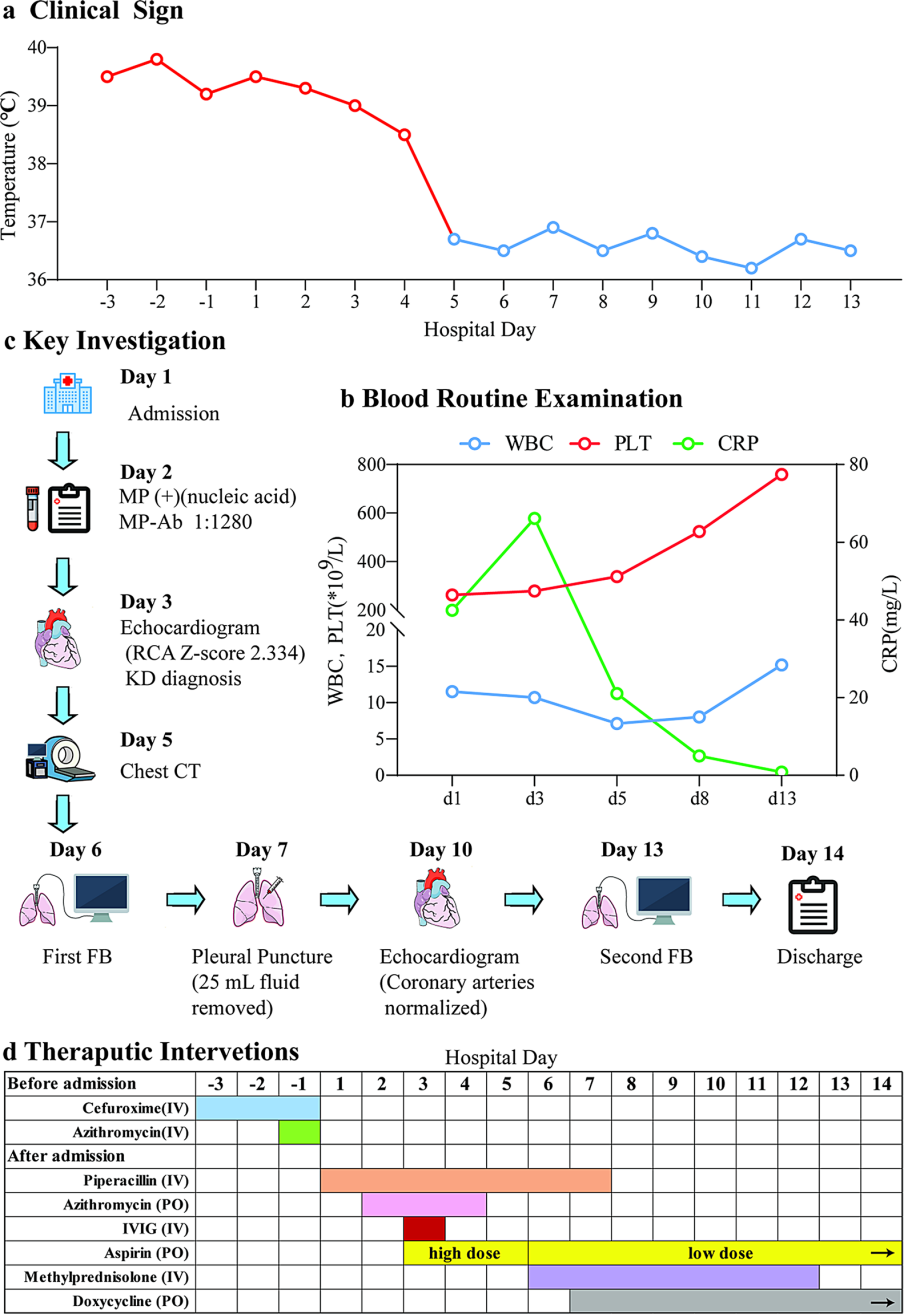
Clinical course and diagnostic timeline of a pediatric patient with concurrent MRMP-associated plastic bronchitis and Kawasaki disease

## Hospital course (Fig. 1)

On Hospital Day 2, laboratory tests revealed a WBC count of 11.51 × 10⁹/L (81.9% neutrophils), a platelet count of 263 × 10⁹/L, and a CRP of 42.5 mg/L. A nucleic acid test for respiratory pathogens was positive for MP, and the MP IgM antibody titer (passive agglutination method) was 1:1280. After confirming MP infection, oral azithromycin was added to the treatment regimen. However, the patient’s condition failed to improve; he remained highly febrile and developed a transient, polymorphous erythematous rash on his trunk.

**Fig. 2:**
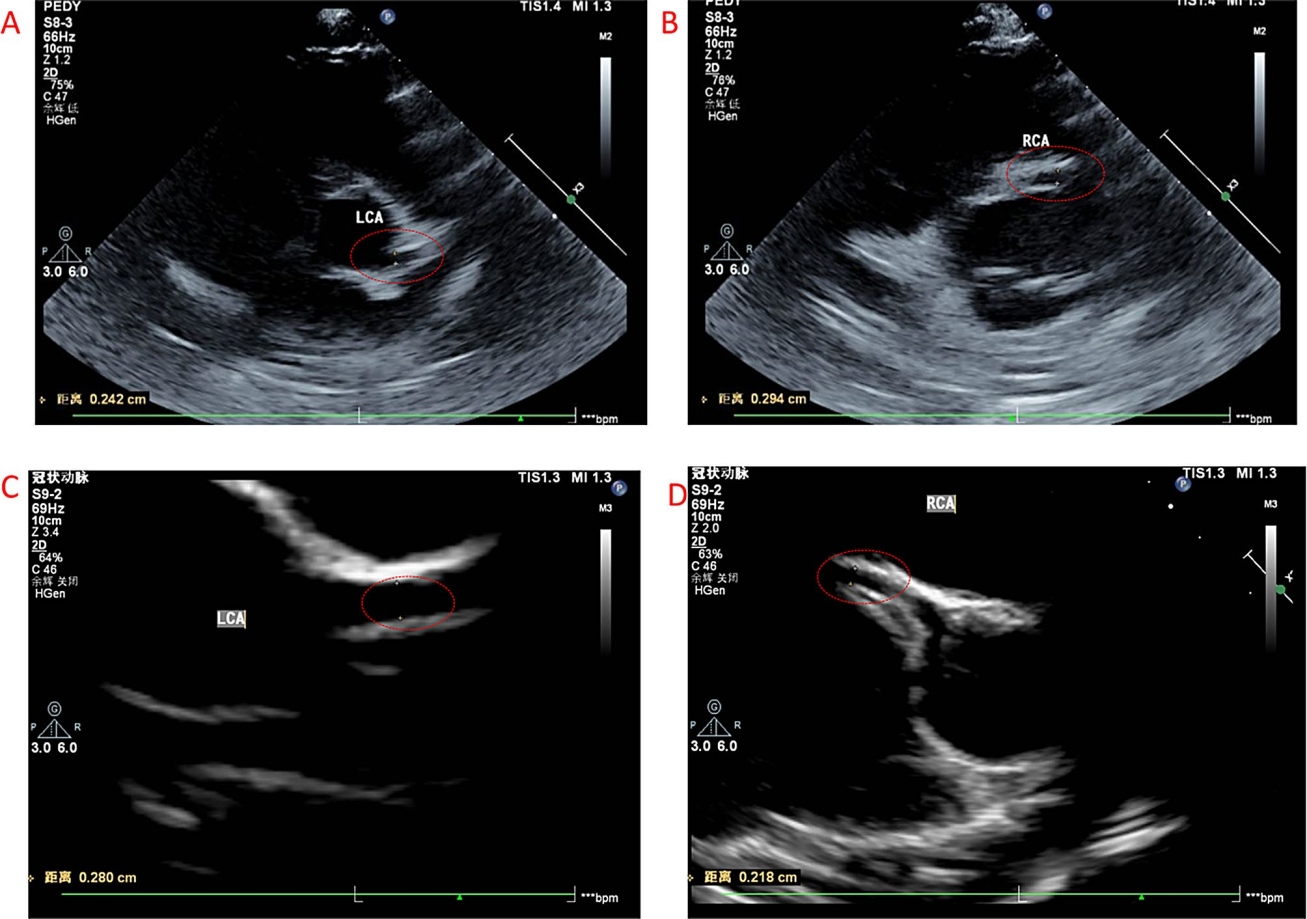
Coronary artery echocardiography before and after treatment for Kawasaki disease. **A** Left Coronary Artery (LCA) before treatment. **B** Right Coronary Artery (RCA) before treatment, showing arterial dilation. **C** Left Coronary Artery (LCA) after treatment. **D** Right Coronary Artery (RCA) after treatment, showing normalization of the vessel diameter

On Hospital Day 3, due to persistent fever and evolving clinical signs, an echocardiogram was performed. It revealed dilation of the right coronary artery (RCA), with the proximal segment measuring 3.0 mm (Z-score + 2.334) and the mid-segment 2.9 mm (Z-score + 2.033) (Fig. 2A, B). Based on these findings, a diagnosis of complete KD was established. The patient fulfilled the principal criterion of fever lasting more than five days, along with four of the five classic clinical criteria: (1) bilateral non-exudative conjunctival injection; (2) changes in the lips and oral mucosa; (3) asymmetric cervical lymphadenopathy (> 1.5 cm); and (4) a polymorphous truncal rash. The coronary artery abnormalities provided critical confirmation of cardiac involvement. Consequently, the patient received a single high dose of intravenous immunoglobulin (IVIG, 2 g/kg; 45 g total) and was started on high-dose oral aspirin (30 mg/kg/day). Laboratory tests on this day showed a CRP elevation to 66.1 mg/L.

On Hospital Day 5, as his temperature gradually stabilized but his cough remained significant, a chest CT was performed to further evaluate the pulmonary inflammation. The scan revealed partial consolidation in both lower lobes, mediastinal lymphadenopathy, and moderate bilateral pleural effusions with adjacent compressive atelectasis (Fig. 4A-B). Notably, there were no imaging signs suggestive of necrotizing pneumonia, such as cavitation or abscess formation. Airway reconstruction indicated multiple bronchial narrowings and suspected mucus plugging, particularly in the lower lobe bronchi.

**Fig. 3:**
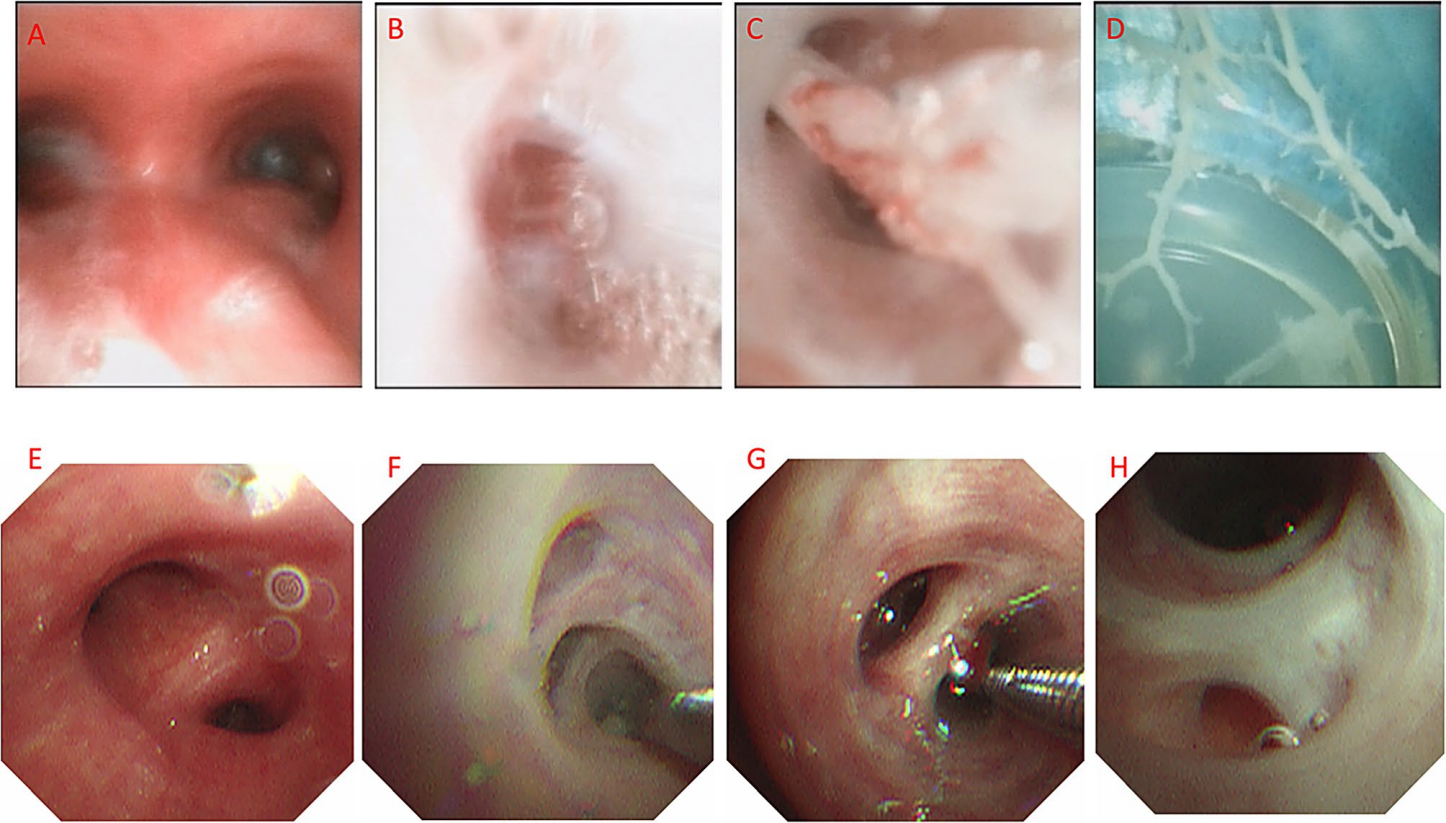
Bronchoscopic findings from two examinations.** A–D** (first bronchoscopy): The bronchial mucosa was markedly hyperemic and edematous (**A**). Necrotic debris and mucus plugs were observed obstructing the segmental bronchi of the left lower lobe (**B**) and right lower lobe (**C**). A large amount of cast-like material was aspirated (**D**). Repeated bronchial brushing, forceps removal, and lavage were performed. Budesonide (1 mg) and N-acetylcysteine (0.3 mg) were locally administered. **E–H** (second bronchoscopy): The bronchial mucosa remained hyperemic and edematous (**E**). Copious flocculent secretions were present in the segmental bronchi of the right lower lobe (**F**) and left lower lobe (**G**), which were repeatedly lavaged. Partial obstruction of the posterior basal segment of the left lower lobe was observed (**H**)

On Hospital Day 6, in light of the CT findings and persistent respiratory symptoms, the patient underwent his first fiberoptic bronchoscopy (FOB). The procedure revealed numerous thick, branching endobronchial casts. Based on these typical findings, a diagnosis of plastic bronchitis (PB) was made (Fig. 3A-D).

The casts, located primarily in the lower lobes, were subsequently removed. Pathological examination of the casts was not performed. The bronchial mucosa underlying the casts appeared hyperemic, edematous, and partially necrotic. Accordingly, methylprednisolone (3 mg/kg/day) was initiated for a planned one-week course, and the aspirin dose was reduced to an antiplatelet level (3–5 mg/kg/day).

On Hospital Day 7, a bedside thoracic ultrasound showed bilateral anechoic, non-septated pleural effusions, with a maximum depth (chest wall to lung) of approximately 4.8 cm on the right and 1.9 cm on the left. A therapeutic thoracentesis was performed on the right side, aspirating 25 mL of yellowish fluid. Pleural fluid analysis confirmed a lymphocyte-predominant exudate; no microbiological culture was performed. On the same day, targeted next-generation sequencing (tNGS) results from the bronchoalveolar lavage fluid (BALF) identified MP as the predominant pathogen (10,008 sequence reads), with co-infection by Streptococcus pneumoniae (7,377 reads) and Staphylococcus aureus (2,267 reads). While no resistance-associated genes were detected for S. pneumoniae or S. aureus, the MP strain was found to harbor the 23S rRNA A2063G mutation, confirming macrolide resistance. After a thorough discussion of the side effects with the family and obtaining informed consent, oral doxycycline was initiated (4 mg/kg/d twice daily for a total of 10 days).

On Hospital Day 10, a follow-up echocardiogram demonstrated complete resolution of the previously noted coronary artery dilation and preserved left ventricular systolic function. The RCA diameter had returned to normal, with the proximal segment measuring 2.4 mm (Z-score + 0.527) and the mid-segment 2.2 mm. The left main coronary artery measured 2.8 mm (Z-score + 0.336), and the left anterior descending artery measured 2.5 mm (Z-score + 1.007) (Fig. 2C-D). No other cardiac structural or functional abnormalities were noted.

On Hospital Day 13, a second FOB (Fig. 3E-H) revealed the disappearance of the casts, although partial bronchial obstruction persisted in the posterior basal segment of the left lower lobe. Laboratory tests showed a WBC count of 15.16 × 10⁹/L, significant thrombocytosis with a platelet count of 759 × 10⁹/L, and a normalized CRP (0.8 mg/L). A follow-up lung ultrasound indicated complete resolution of the pleural effusions.

On Hospital Day 14, the patient was afebrile and clinically stable and was therefore discharged with instructions to complete the course of oral doxycycline and continue low-dose aspirin.

### One-month follow-up

**Fig. 4:**
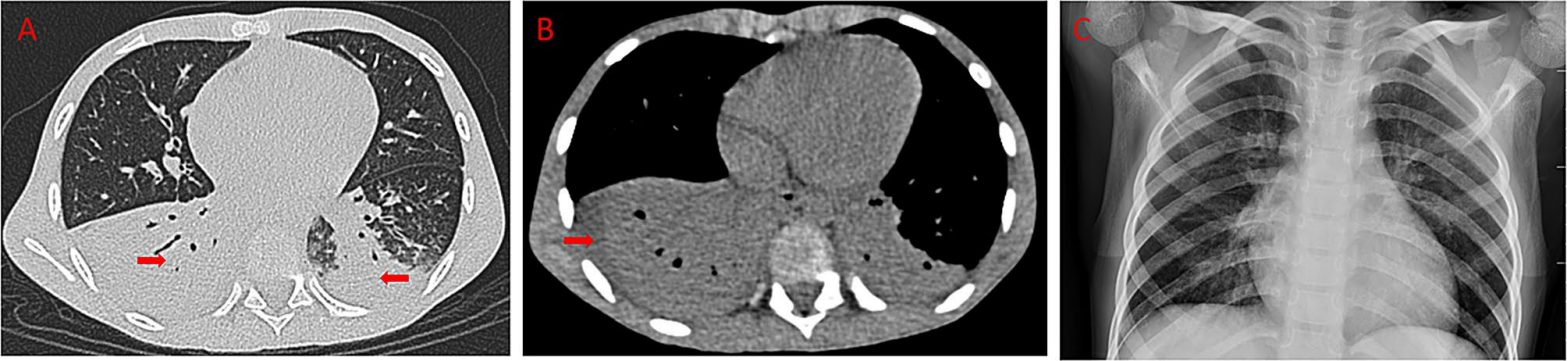
Chest imaging before and after treatment. **A**: Pre-treatment chest CT (lung window) shows partial consolidation in the lower lobes of both lungs (as indicated by red arrows). **B**: Pre-treatment chest CT (mediastinal window) reveals moderate bilateral pleural effusion and partial compressive atelectasis of the adjacent lower lobes (as indicated by red arrows). **C**: CXR at 1-month follow-up after discharge demonstrates resolution of pulmonary inflammation with no new lesions

At the one-month follow-up visit, the patient was asymptomatic with stable respiratory function. A repeat CXR (Fig. 4C) showed significant resolution of the previously observed parenchymal opacities, and a follow-up echocardiogram confirmed that all coronary artery dimensions remained within the normal range.

### Final discharge diagnoses

Severe Pneumonia due to:

Macrolide-resistant Mycoplasma pneumoniae

Streptococcus pneumoniae

Staphylococcus aureus

Plastic Bronchitis (PB)

Complete Kawasaki Disease (KD)

Bilateral Pleural Effusion

## Discussion

We report the first case of confirmed MRMP infection complicated by both PB and KD in a pediatric patient. This case highlights the potential for MRMP to trigger unprecedented multisystem complications in the antibiotic resistance era, expanding our understanding of Mycoplasma-associated disease manifestations.

### Epidemiological context and literature review

The global prevalence of MRMP has increased substantially in recent years. A 2024 Beijing survey demonstrated significantly elevated M. pneumoniae detection rates among children with pneumonia, with the A2063G mutation representing over 99% of resistant strains [5]. Notably, among 126 children with A2063G-positive MRMP pneumonia, 25.4% developed plastic bronchitis [11]. These findings align with data from Ohio, USA, and Taiwan, confirming the resurgence of pediatric MRMP infection with persistently high resistance rates [12, 13].

Current mechanistic understanding of MRMP complications focuses predominantly on individual disease processes. For plastic bronchitis, the pathogenesis involves multiple converging pathways. The Community-Acquired Respiratory Distress Syndrome (CARDS) toxin—a unique M. pneumoniae exotoxin—directly damages airway epithelial cells, disrupts barrier integrity, and induces type I immune responses, triggering sustained airway inflammation and mucus hypersecretion [14, 15]. Concurrently, M. pneumoniae infection activates neutrophils and macrophages to release pro-inflammatory cytokines while activating coagulation cascades, promoting inflammatory exudate accumulation and fibrin deposition that culminate in bronchial cast formation [16].

For KD, emerging evidence implicates microbial infections in systemic immune vasculitis pathogenesis. A 2024 retrospective study found that KD patients with concurrent M. pneumoniae infection exhibited more severe cardiac involvement and higher hepatic dysfunction rates, suggesting M. pneumoniae may act as a synergistic trigger or disease modifier [17]. Multiple case reports support this hypothesis, proposing that M. pneumoniae activates T cells through superantigen mechanisms, participating in KD pathogenesis [18].

### Pathophysiological considerations

The concurrent presentation of PB and KD in our patient raises important mechanistic questions. No studies have directly investigated shared pathogenic mechanisms between these two conditions. Based on our case’s characteristics, we identified three potentially interconnected factors: first, the A2063G mutation-mediated macrolide resistance enabled persistent infection [19]; second, tNGS revealed polymicrobial infection with M. pneumoniae (10,008 sequence reads), Streptococcus pneumoniae (7,377 reads), and Staphylococcus aureus (2,267 reads), correlating with a dramatic CRP rise from 9.6 mg/L to 66.1 mg/L within 3 days (689% increase); third, despite distinct pathogenic mechanisms, both PB and KD involve excessive inflammatory responses and immune dysregulation [14, 17].

We propose that sustained polymicrobial infection triggered shared inflammatory dysregulation pathways, manifesting locally as airway cast formation and systemically as vascular endothelial dysfunction. This hypothesis requires validation through future studies incorporating detailed immunological profiling and cytokine analyses.

### Therapeutic approach and outcomes

Despite the high prevalence of MRMP in China (>90%), our initial use of macrolide antibiotics aligns with recommendations from the “Expert Consensus on the Diagnosis and Treatment of Macrolide-Resistant Mycoplasma pneumoniae Pneumonia in Children” (2024) [2]. Even in high-resistance regions, this consensus supports macrolides as first-line therapy for mild to moderate cases, based on their excellent safety profile, pulmonary tissue penetration, and potential anti-inflammatory and immunomodulatory effects. The consensus recommends considering alternative antibiotics when symptoms do not significantly improve within 48–72 h. In our patient, after initial azithromycin therapy failed and tNGS confirmed the A2063G mutation, we promptly switched to doxycycline. Although tetracycline use in children under 8 years remains controversial, recent 2024 studies demonstrated that short-term (7–10 days) tetracycline therapy is safe and effective for severe MRMP pneumonia [20]. Doxycycline’s lower minimum inhibitory concentration effectively suppresses M. pneumoniae replication, reducing bacterial burden and consequently decreasing CARDS toxin production and airway inflammation [14, 15], ultimately contributing to favorable clinical outcomes.

For PB management, we employed therapeutic bronchoscopy combined with corticosteroid therapy. Two bronchoscopic procedures successfully removed bronchial casts while delivering topical budesonide and N-acetylcysteine to improve airway inflammation and mucus clearance. Systemic methylprednisolone downregulated pro-fibrotic mediators including TGF-β and IL-6, inhibiting fibroblast proliferation and preventing airway remodeling [21].

KD treatment followed standard protocols with high-dose intravenous immunoglobulin (IVIG, 2 g/kg) and aspirin. IVIG exerts therapeutic effects through multiple mechanisms: competitive Fcγ receptor binding, superantigen neutralization, and immune complex clearance, thereby correcting T/B cell dysregulation and suppressing systemic inflammation [22]. A 2025 retrospective study demonstrated that early adjunctive corticosteroid therapy in high-risk KD patients reduces coronary artery lesion progression [23]. Following combined IVIG and corticosteroid therapy, our patient experienced rapid defervescence, normalized inflammatory markers, and complete resolution of coronary artery dilation, validating this comprehensive therapeutic strategy.

### Clinical implications

This case yields several important clinical insights. First, in MRMP-endemic regions, clinicians should maintain heightened suspicion for resistant strains in children with macrolide-refractory pneumonia, performing early molecular diagnostics such as tNGS to guide precision antimicrobial therapy [2]. Second, MRMP infection can trigger severe multisystem complications beyond conventional disease associations; clinicians must actively screen for pulmonary complications (e.g., PB) and extrapulmonary manifestations (e.g., KD) in patients presenting with persistent fever, progressive respiratory symptoms, or mucocutaneous findings [21]. Third, bronchoscopy remains indispensable for PB diagnosis and treatment, enabling definitive diagnosis, therapeutic cast removal, and guidance of subsequent management [11]. Finally, individualized combination therapy—incorporating appropriate alternative antibiotics, corticosteroids, and IVIG—is essential for optimizing outcomes in severe MRMP-associated immune-mediated complications.

## Limitations

This study has several inherent limitations. As a single case report, our findings cannot be generalized to broader patient populations. The lack of control groups and mechanistic studies prevents us from establishing causal relationships. Furthermore, pathological examination of the bronchial casts was not performed in this case. Additionally, the relatively short follow-up period (1 month) hindered assessment of long-term outcomes, particularly regarding risks of bronchiolitis obliterans and cardiovascular sequelae that may appear later. Finally, comprehensive immunological analysis was not conducted, limiting our ability to elucidate common immune dysregulation pathways that could potentially lead to both plastic bronchitis and KD in the context of MRMP infection.

### Future directions

Future investigations should pursue the following objectives: First, multicenter prospective cohort studies are needed to systematically characterize the incidence, clinical features, risk factors, and prognosis of PB and KD complicating MRMP infection. Second, comprehensive immunological profiling (cytokine arrays, T cell subset analyses, neutrophil extracellular trap quantification) and transcriptomic studies should elucidate whether shared immune dysregulation pathways underlie PB and KD in the MRMP infection context [24]. Third, randomized controlled trials should optimize treatment protocols, defining optimal alternative antibiotic selection and corticosteroid/IVIG timing and dosing strategies [20, 23]. Fourth, long-term follow-up cohorts (≥ 2–5 years) should assess risks of bronchiolitis obliterans, chronic pulmonary function impairment, and cardiovascular complications, informing evidence-based surveillance protocols.

## Data Availability

The data can be obtained from the corresponding authors upon reasonable request.

## Abbreviations

BALF: Bronchoalveolar lavage fluid
CARDS: Community-Acquired Respiratory Distress Syndrome (toxin)
CAP: Community-acquired pneumonia
CRP: C-reactive protein
CT: Computed tomography
CXR: Chest X-ray
ENT: Ear, nose, and throat
FOB: Fiberoptic bronchoscopy
IgM: Immunoglobulin M
IVIG: Intravenous immunoglobulin
KD: Kawasaki disease
MIC: Minimum inhibitory concentration
MP: Mycoplasma pneumoniae
MRMP: Macrolide-resistant Mycoplasma pneumoniae
PB: Plastic bronchitis
RCA: Right coronary artery
tNGS: Targeted next-generation sequencing
WBC: White blood cell
